# Influence of genetic factors in elbow tendon pathology: a case-control study

**DOI:** 10.1038/s41598-020-63030-7

**Published:** 2020-04-16

**Authors:** Yasser Alakhdar Mohmara, Jill Cook, Josep C. Benítez-Martínez, Emily R. McPeek, Antonio Alberola Aguilar, Emilio Soria Olivas, Sergio Hernandez-Sanchez

**Affiliations:** 1 Department of Physiotherapy, Universitat de Valencia, Valencia, Spain; 2 La Trobe University, Community and Allied Health, Melbourne, Australia; 3 Department of Physiology, Universitat de Valencia, Valencia, Spain; 4 Intelligent Data Analysis Laboratory, E.T.S.E., University of Valencia, Valencia, Spain; 5 Center for Translational Research in Physiotherapy. Physiotherapy area. Miguel Hernandez University, Elche, Spain

**Keywords:** Orthopaedics, Predictive markers, Genetics research, Risk factors

## Abstract

Elbow tendinopathy is a common pathology of the upper extremity that impacts both athletes and workers. Some research has examined the genetic component as a risk factor for tendinopathy, mainly in the lower limbs. A case-control study was designed to test for a relationship between certain collagen gene single nucleotide polymorphisms (SNPs) and elbow tendon pathology. A sample of 137 young adult athletes whose sports participation involves loading of the upper limb were examined for the presence of structural abnormalities indicative of pathology in the tendons of the lateral and medial elbow using ultrasound imaging and genotyped for the following SNPs: COL5A1 rs12722, COL11A1 rs3753841, COL11A1 rs1676486, and COL11A2 rs1799907. Anthropometric measurements and data on participants’ elbow pain and dysfunction were collected using the Disabilities of the Arm, Shoulder and Hand and the Mayo Clinic Performance Index for the Elbow questionnaires. Results showed that participants in the structural abnormality group had significantly higher scores in pain and dysfunction. A significant relationship between COL11A1 rs3753841 genotype and elbow tendon pathology was found (p = 0.024), with the CT variant associated with increased risk of pathology.

## Introduction

Tendinopathy is a common condition that affects a large portion of the population, making up 30% of all musculoskeletal injuries^1^ and being the most prevalent tendon disorder^2^. It is generally defined as an overuse injury resulting in tendon degeneration after a failed early inflammatory healing response^3^, leading to collagen disorientation and disorganization in the absence of classic inflammatory changes, accompanied by pain and dysfunction^4,5^. Tendon homeostasis relies on a dynamic remodeling process influenced by tendon loading and cytokines, among other things. Evidence support the expression and functional involment of pro-inflammatory cytokines such as interleukin-1ß in tissues surrounding mechanically injured tendon^6^. Tendon inflammation and tendinopathy are possible manifestations of a disturbance of this homeostasis. The role of inflammation in tendon healing or failure to heal is complex and not fully understood, but it is known that an initial inflammatory response is required to begin the healing process. On the other hand, cytokines appear to be capable of inducing a failed healing response in several diseases of connective tissue (cartilage, bone, synovial joint) possibly by altering the expression of matrix metalloproteinases^7^. Probably the tendon is no exception, although there are no conclusive data at present on the role of these cytokines in the tendinopathy development^6,7^.

The exact incidence of elbow tendinopathy is unknown, but it is estimated to affect 1–3% of adults each year in the lateral elbow^8^ and 0.1–0.75% in the medial elbow^9^. It has been reported that the pathogenesis of the medial pathology parallels that of the lateral pathology, with similar patterns of angiofibroblastic degeneration^10^. In athletes, individuals who practice sports that require overhead throwing or repeated forearm pronation and wrist flexion, such as baseball and swimming, represent a group with elevated risk, especially when those movements are combined with a so-called “power grip”, as in tennis and golf^4,8^. However, elbow tendinopathy is not limited to athletes and can occur in many routine and work-related activities that involve similar movements^5^.

Due to differential responses to the same external load, research on the topic of intrinsic risk factors in tendinopathy has increased in recent years, which in other studies have included anthropometric factors such as age^11^ (peak incidence of tennis elbow occurs at age 35–55 years) and obesity^12^, as well as genetic factors. To date, several studies have described the role of genetic factors in Achilles and patellar tendinopathies^13^, but studies involving the tendons of the upper limbs are scarce, with just a few studying genetic factors in rotator cuff tendinopathy and only one study on tennis elbow^14^.

Collagen is the principal component of the tendon extracellular matrix (ECM), and its function is related to the formation of fibril and microfibril substances in the ECM, playing an important role in determining the specific properties of each tissue^15^. Type I collagen is the most abundant in tendons^1^, although variable amounts of types II, III, V and XI can also be found^16^. While type III collagen is the main type involved in regulation of fibrillogenesis and tendon extensibility, the minor fibrillary types V and XI are associated with types I and II, respectively, determining their quantity and quality^17^. The primary isoform of type V is a heterotrimer consisting of two α1 chains (encoded by COL5A1 gene) and one α2 chain (encoded by COL5A2 gene), which intercalates with type I collagen and modulates fibrillogenesis. The primary isoform of type XI is a heterotrimer consisting of an α1 chain and an α2 chain (COL11A1 and COL11A2, respectively), plus an α2 chain from type II; these molecules form strong crosslinks between tendon cells and also help maintain the spacing and diameter of type II collagen fibrils.

Certain mutations in collagen synthesis genes have been associated with disorders resulting from alteration or loss of collagen function. Several forms of Ehlers-Danlos syndrome are caused by mutations in COL1A1^18^, while hundreds of mutations in COL3A1 have been found to cause the vascular type of the disease^19^. Mutations in COL5A1 and COL11A1 are seen in certain types of Ehlers-Danlos syndrome and Marshall syndrome^20^, respectively. Mutations in COL11A2 have been associated with otospondylomegaepiphyseal dysplasia^21^ and Weissenbacher-Zweymüller syndrome^22^. Both COL11A1 and A2 have been associated with Stickler syndrome^23^. All of these disorders can impact joint extensibility or cause collagen-related skeletal abnormalities.

As the main component of the tendon ECM, changes in collagen and genes governing its synthesis and degradation have been promising objects of research on tendinopathy etiology and risk factors. With regard to the major fibrillary types, increased expression of COL1A1 and COL3A1 genes has been found in tendinopathic tendons^24^. In the search for genetic risk factors, the alleles of COL5A1 SNP rs12722 (C and T) have been previously studied^25,26^. While genotype CC has been associated with lower risk of chronic Achilles tendinopathy^12^, genotype CT is associated with less elasticity in the lower limb^27^, and genotype TT is associated with lower range of mobility, particularly in elderly subjects^28^, and greater risk of chronic Achilles tendinopathy^29^. Altinisik *et al*. (2015) found a significant association between COL5A1 rs12722 and rs13946 variants and risk of lateral epicondyle tendinopathy (tennis elbow); the authors reported a protective effect of the CC genotype in development of elbow tendinopathy for SNP rs12722, and of the TT genotype for SNP rs13946^14^. However, some other studies have failed to find an independent association^29^.

COL11A1 and A2 variants have also been studied in relation to tendinopathy, although to a lesser extent. Hay *et al*. (2013) analyzed COL11A1 rs3753841 (T/C), COL11A1 rs1676486 (C/T) and COL11A2 rs1799907 (T/A) to establish their independent association with chronic Achilles tendinopathy without being able to establish such a relationship. However, they did report an overrepresentation in the tendinopathy group of certain inferred pseudohaplotypes from the combination of these polymorphisms, as well as pseudohaplotypes of the collagen XI polymorphisms in combination with COL5A1 rs7174644 (-/AGGG) genotype^30^. Finally, a more recent line of investigation has been epigenetic risk factors for tendinopathy, particularly DNA methylation and its effects on expression levels of genes regulating collagen and other components of the ECM^31^.

Considering the existing literature on the relationship between genetic factors and tendinopathy, this study aims to examine the association of different SNP genotypes with structural abnormalities indicative of elbow tendon pathology, namely COL5A1 rs12722, COL11A1 rs3753841, COL11A1 rs1676486 and COL11A2 rs1799907, selected in order to compare results with previous studies on patellar^24,31^, Achilles^13,25^ and elbow tendinopathies^14^.

## Results

The tendon pathology group as determined by abnormal tendon structure on ultrasound imaging was also found to have DASH sports and occupational scores that were significantly higher than those of the normal group (p = 0.03 and p = 0.005, respectively), reflective of higher perceived pain and lower perceived functionality. A chi-square test revealed a statistically significant association between the COL11A1 rs3753841 genotype and elbow tendon pathology (p = 0.024). No significant associations were found for the other SNPs studied.

Allele frequency for COL11A1 rs3753841 in the sample population was 65.69% cytosine/34.31% thymine for the first allele, and 16.79% cytosine/83.21% thymine for the second allele. The genotype distribution in the sample population was 16.79% CC, 48.91% CT, 34.31% TT (Table 1). Participants with genotype CT had tendon pathology with greater frequency than the two homozygous genotypes.

**Table 1:** Genotype Frequencies For SNPs Studied.

	Cases (n = 36)	Controls (n = 101)	p
Genotype COL5a1 rs12722, n (%)			0.844
C/C	4 (11.1)	8 (7.9)	
C/T	20 (55.6)	58 (57.4)	
T/T	12 (33.3)	35 (34.7)	
Genotype COL11a1 rs3753841, n (%)			0.024*
C/C	2 (5.6)	21 (20.8)	
C/T	24 (66.7)	43 (42.6)	
T/T	10 (27.8)	37 (36.6)	
Genotype COL11a1 rs1676486, n (%)			0.862
C/C	29 (80.6)	77 (76.2)	
C/T	6 (16.7)	21 (20.8)	
T/T	1 (2.8)	3 (3.0)	
Genotype COL11a2 rs1799907, n (%)			0.807
A/A	4 (11.1)	9 (8.9)	
A/T	11 (30.6)	27 (26.7)	
T/T	21 (58.3)	65 (64.4)	

C = cytosine, T = thymine, A = adenine; *significant, p < 0.05.

Other intrinsic factors were significantly associated with the presence of elbow tendinopathy, most notably higher values for BMI (p = 0.00002) and related variables, such as percent body fat (p = 0.00003), weight (p = 0.001), and waist circumference (p = 0.003). There were no significant differences between the two groups in age or gender composition. In the logistic regression model, the relationship between the COL11A1 rs3753841 SNP genotype ceased to be significant when controlling for BMI.

**Figure 1:**
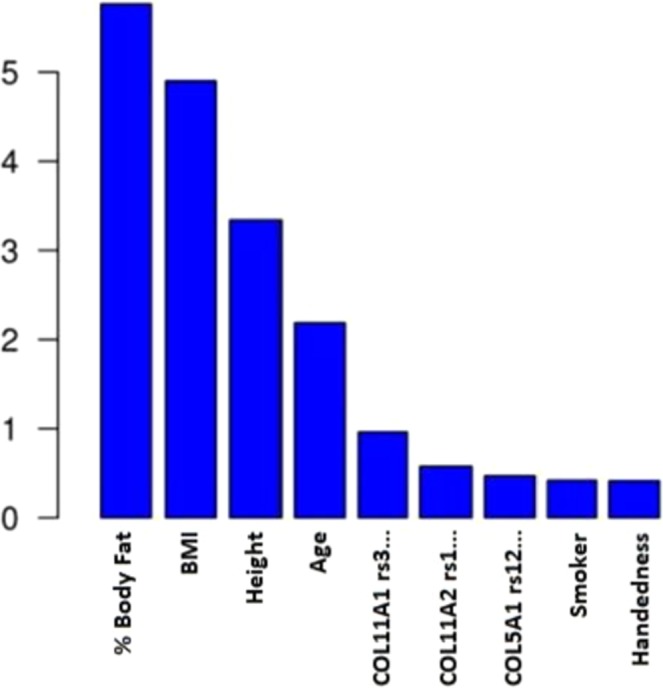
Relative importance of intrinsic variables in elbow tendon pathology according to the random forest test.

Based on the results of the random forest test (Fig. 1), the COL11A1 rs3753841 SNP genotype had the greatest influence of the genetic variants studied, followed by COL11A2 rs1799907 genotype and COL5A1 rs12722 genotype. Anthropometric factors were generally found to be more statistically important than genetic factors.

## Discussion

In this study, the genotype for SNP COL11A1 rs3753841 was associated with incidence of elbow tendon pathology as diagnosed through ultrasound imaging; participants in the pathology group were significantly more likely to have the CT genotype. Genotype distributions for COL11A1 rs3753841 are comparable to those obtained by Hay *et al*.^26^, who reported 16.4% CC, 44.4% CT, 39.2% TT, comparable to our values of 16.79% CC, 48.91% CT, and 34.31% TT. These values are also within the Hardy-Weinberg equilibrium (HWE) expected values (χ^2^ = 0.01).

COL5A1 rs12722 has been associated with lateral elbow tendinopathy (tennis elbow) in a previous study^14^. However, we found no significant relationship with respect to this genotype. This could be due to the fact that our study considered both medial and lateral elbow tendons. Our diagnostic criteria were also different, as Altinisik *et al*. relied on clinical criteria that did not involve imaging through ultrasound or any other means. With our criteria, we were able to remove the subjectivity inherent in patients’ reporting of symptoms when sorting participants into groups. While this may not reflect the typical procedure in real-life clinical practice, imaging is the only method to confirm tendinopathy as symptoms are variably associated with pathology^32^, and such a comparatively objective measure of pathology is advantageous in the search for risk factor relationships.

One of the limitations of this study has been the sample size resulting from financial limitations given the high cost of genetic testing, although similar sample sizes have been used by other authors^23,33,34^. Genotype frequencies for both COL11A1 rs1676486 and COL11A1 rs3753841 were well within HWE expected values, while there was a departure from HWE with COL5A1 rs12722 and COL11A2 rs1799907. For COL5A1 rs12722, our frequencies were comparable to those of some previous authors^29^. However, our values for COL11A2 rs1799907 were quite different from previous studies^26^. Thus, it is hard to say with certainty that the departures from HWE are due to a small sample size. Likewise, it is possible that the difference in genotype frequencies between studies are due to ethnic variances between study populations.

Another limitation is that a significant association was found between elbow tendon pathology and several related anthropometric measurements, namely BMI, percent body fat, weight and waist circumference. We must acknowledge a potentially confounding effect of these variables, given that a logistic regression returned a non-significant relationship between the SNP of interest and tendon pathology when controlling for BMI. In the future, it could be interesting to investigate the relationship of these variables in greater depth. Given the small amount of fat stored locally around the elbow, this may also provide insight into the mechanisms by which BMI influences tendon pathology. It has been proposed that hormonal imbalances and low-grade inflammation resulting from obesity may contribute to failed tendon healing and eventual degeneration^35^; since our participants were healthy athletes and not obese, it could be interesting in future work to analyze their blood samples for hormonal imbalances or the presence of pro-inflammatory cytokines, and investigate potential relationships between those factors and elbow tendinopathy as well as between those factors and anthropometric variables, such as percent body fat.

To our knowledge, this is this first study reporting on the influence of COL11A1 rs3753841 genotype in the context of elbow tendon pathology. Our study presents new potential lines of research, including a more complete analysis of genetic factors related to collagen regulation and other components of the tendon extracellular matrix as they relate to elbow tendinopathy.

Potential genetic predisposition could serve as another tool for clinicians to be used in combination with family history, personal history of other tendon injuries, and level of participation in high-risk sports. A more thorough understanding of intrinsic risk factors could be a helpful tool in establishing preventive measures and developing individualized protocols in both treatment and training to minimize risk of this type of injury.

## Methods

A sample of 137 participants (mean age 23 ± 5.5 years, 77 men and 60 women) were selected from the general population based on their participation in sports that involve loading of the upper limb, recruited through information sessions at the Faculty of Physiotherapy, Faculty of Sciences of Physical Activity and Sport, and through meetings with different sports teams.

Inclusion criteria for the study were: age 18–50 years, weekly participation a sport involving the upper limb ≥ 4 hours, and continuous participation in that sport ≥ 2 years. Volunteers were excluded in the case of previous surgery or osteoarthritis of the elbow or shoulder, history of elbow subluxation, dislocation, or fracture of the humerus, radius or ulna, treatment for shoulder problems, cancer, medications known to affect tendon characteristics in the past 6 months, pregnancy, or inflammation of the elbow such as rheumatoid arthritis or ankylosing spondylitis.

The study protocol was approved by The Human Research Ethics Committee of the University of Valencia (H1409657453224) in accordance with the principles established in the Declaration of Helsinki, the Council of Europe Convention, and Spanish law regarding biomedical research, protection of personal data, and bioethics. All participants were informed of the purpose of the study, its potential benefits and risks or inconveniences that could result from the study protocol, and signed the informed consent form they were provided.

A history of each participant’s lifestyle habits, clinical history, sports participation and family history was collected. Participants completed the Disabilities of the Arm, Shoulder and Hand (DASH) general, sports and occupational questionnaires, and the Mayo Clinic Performance Index for the Elbow questionnaire to quantify levels of pain and dysfunction^10,36^. Anthropometric data including height, weight, waist circumference, and body fat percentage as measured by bioelectric impedance were also collected.

Participants were distributed into two groups according to the presence or absence of pathology in any of the tendons of the elbow. For this purpose, a comprehensive ultrasound examination (FUJIFILM SonoSite NanoMax US system) performed by an experienced musculoskeletal physiotherapist specialized in ultrasound assessment was used as an objective measure to evaluate the integrity of the tendons of the elbow. Tendons were classified as pathological if they presented an increase in diameter relative to normal, hypoechogenicity and/or evidence of partial or total tears. Based on the results of the ultrasound examination, 36 participants were finally assigned to the case group, and 101 to the control group. Participant characteristics are shown in Table 2.

**Table 2:** Characteristics of Tendon Pathology Cases and Controls.

	Cases (n = 36)	Controls (n = 101)
**Sex, n (%)**		
Male	18 (50.0)	59 (58.4)
Female	18 (50.0)	42 (41.6)
Age, mean ± SD (range)	23.1 ± 4.7 (18–35)	23.1 ± 5.8 (18–44)
**Dominant hand, n (%)**		
Right	32 (88.9)	92 (91.1)
Left	2 (5.6)	6 (5.9)
Ambidextrous	2 (5.6)	3 (3.0)
BMI, mean ± SD (range)	24.7 ± 3.6 (20.5–36.5)	21.9 ± 2.8 (17.0–30.9)
**Side of tendinopathy, n (%)**		
Dominant only	20 (55.6)	N/A
Non-dominant only	5 (13.9)	N/A
Bilateral	11 (30.6)	N/A
**Affected tendon, n (%)**		
Lateral	10 (27.8)	N/A
Medial	16 (44.4)	N/A
Combination	10 (27.8)	N/A

For genotype analysis, a blood sample of approximately 4.5 ml was extracted via venipuncture from the forearm vein in Vacutainer EDTA tubes, stored at 4 °C during the experimental protocol and later moved to −80 °C storage, where samples were stored for two months. Once the data collection phase was finished, all samples were moved under optimal conditions to the Genetics and Molecular Biology Unit at La Ribera University Hospital in Alzira (Valencia), where the genetic analysis was performed.

DNA was extracted using the standard procedures. To determine the genotype of the selected polymorphisms in the blood samples, a TaqMan SNP genotyping analysis (Applied Biosystems; Foster City, California, United States) was performed using the Real Fast 7900HT real-time PCR system (Applied Biosystems). Genotype results were reproduced for each participant in triplicate in independent tests.

The mathematics software R was used for data analysis in this study. A chi-square test was used to test for associations between categorical variables, including the different genotypes, and the presence of tendon pathology. For quantitative variables, a Wilcoxon-Mann-Whitney test was used to find significant differences between the normal and pathological groups. A logistic regression was also used to control for certain intrinsic variables that have been reported to have an association with tendon pathology, such as age and BMI, as well as sex, in order to account for potentially confounding effects of these variables when determining a possible association between SNP genotypes and pathology. A random forest test, a type of machine learning statistical model, was used to assess the relative importance of each tested variable^37^.

## Conclusions

In the present study, the genotype for SNP COL11A1 rs3753841 was associated with elbow tendon pathology; subjects in the pathology group were significantly more likely to have the CT genotype. None of the other SNPs studied showed a significant association. Significant relationships were also found between the anthropometric variables BMI, percent body fat, and waist circumference and elbow tendon pathology.

## Practical Implications

- A significant relationship was found between the genotype of COL11A1 rs3753841 and structural abnormality in elbow tendons.
- A significant relationship was found between structural abnormality in elbow tendons and body mass index, as well as percent body fat and waist circumference.
- Participants with elbow tendons classified as pathological using ultrasound imaging reported significantly higher levels of elbow pain and dysfunction.
- Those with the risk variant who are overweight should be encouraged to lose weight, as there appears to be a genotype-BMI interaction.
